# Misdiagnosed male breast cancer with an unknown primary tumor: A case report

**DOI:** 10.3892/ol.2014.2111

**Published:** 2014-05-05

**Authors:** WEN-WU WANG, LANG CHEN, XUE-NONG OUYANG

**Affiliations:** Department of Medicine Oncology, Fuzhou General Hospital of Nanjing Military Command, Fuzhou, Fujian 350025, P.R. China

**Keywords:** misdiagnosis, male breast cancer, primary tumor

## Abstract

Compared with female breast cancer, male breast cancer (MBC) has an extremely low morbidity, later staging and fewer breast tissues. The lumps are easier to invade in the center and the majority of the cases are positive for metastatic lymph node, with the typical clinical manifestation as a painless mass in partial breast. MBC with an unknown primary tumor is rare and is often prone to misdiagnosis, resulting in a delay in correct treatment. Such a case is extremely significant for clinical reference. The current study presents a 58-year-old male who developed a painless mass in the left armpit and received armpit mass biopsy and pathological examination which showed glandular cancer, with a high possibility of mammary primary tumor. The patient was administered four cycles of paclitaxel plus oxaliplatin chemotherapy. However, three months later, the patient identified novel disseminated lymph nodes in the left armpit. The initial pathological section and paraffin blocks were re-examined and the patient was finally diagnosed with breast invasive ductal carcinoma based on the metastases pathology and immunohistochemical examination. No breast mass was found on physical examination of the patient and the tumor markers, including cancer antigen 125 and carcinoembryonic antigen, were normal. No primary tumors were observed in the mammography and PET-CT and the primary tumor was not found following the left breast modified radical mastectomy.

## Introduction

Male breast cancer (MBC) is a rare and special type of breast cancer, which is rarely observed in clinical practice. The incidence of MBC is ~1% among all breast cancer patients (1), however, recent studies in the USA have shown that the incidence of MBC is on the rise (2). Since MBC is rarely observed in clinical practic, few prospective and randomized clinical studies have analyzed the disease. In addition, due to the rarity of MBC, patients and physicians are less suspicious of the disease, which therefore, delays diagnosis to a great extent and leads to the development of illness. Furthermore, the prognosis of such a condition has not been significantly improved over the past 25 years (3). MBC with an unknown primary lesion is even rarer and presents a special type of breast cancer of which the initial symptom is axillary lymphatic metastasis. However, the primary lesion within the breast can not be detected during physical examination and imaging examination. MBC was first described Halsted (4) in 1907. The main treatment for MBC is extended resection of the suspicious breast lesions in combination with axillary lymph node dissection, which attack the root causes of the disease and improve patients quality of life. In addition, the adjuvant chemotherapy regimen is likely to be administered according to the standard used for female breast cancer (5). MBC with an unknown primary tumor is rare and therefore prone to misdiagnosis, which results in treatment delay. Such cases are extremely significant for clinical reference and the current study presents a case of a clinically misdiagnosed MBC with an unknown primary tumor.

## Case report

The current study presents the case of a 58-year-old male who developed a painless mass in the left armpit in May 2009. The mass was hard and 0.8×0.6 cm in size. There was no obvious discomfort and the mass was treated with drugs, including amoxicillin capsules (500 mg, every 8 h for five days). In May 2011, the mass had become significantly larger and was accompanied by pain. The patient received anti-infection treatment with ceftriaxone sodium at the Fujian Union Hospital (Fuzhou, China) and an armpit mass biopsy was obtained. The pathological examination showed glandular cancer with a high possibility of a mammary primary tumor. For further anticancer treatment, the patient was moved to the larger and specialized Fujian Cancer Hospital (Fuzhou, China), where a metastatic poorly-differentiated cancer was considered following the pathology consultation, and the primary tumor was unknown. It was recommended that the patient should have a positron emission tomography/computed tomography (PET-CT) general physical check-up, however, the primary tumor remained unknown. The final diagnosis was of secondary lymph node cancer, with an unknown primary tumor. The patient was empirically administered four cycles of paclitaxel (200 mg) plus oxaliplatin (120 mg) chemotherapy between June and October 2011 and subsequently the symptom was eased. The curative effect was evaluated as a partial response, and the patient was later discharged.

Three months after discharge, the patient found new disseminated lymph nodes in the left armpit. On January 9, 2012, the patient came to Fuzhou General Hospital of Nanjing Military Command (Fuzhou, Fujian, China) and the admission examination showed that changes to the mass in the left armpit were visible. Several enlarged lymph nodes, ~1.2×0.5 cm in size, were palpable, hard and fixed in position, with a complete surface. Pain was felt upon the application of light pressure. The superficial lymph nodes were not enlarged and the heart, lungs and abdomen showed no obvious abnormalities. A PET-CT examination was also carried out and the results revealed that higher metabolism occurred in the lymph nodes in the left armpit. The malignant tumor, along with tumor activity, was considered, and again the diagnosis was of secondary lymph node cancer, with an unknown primary tumor. Between March 26 and May 26, 2012, the patient was administered docetaxel (100 mg) plus lobaplatin (80 mg) chemotherapy. The chemotherapy continued smoothly and no obvious adverse reactions occurred. Following three cycles of chemotherapy, the curative effect was evaluated as progressive disease. On June 9, 2012, the patient returned for treatment again. The disease had not been effectively controlled by the two chemotherapy schemes (200 mg paclitaxel plus 120mg oxaliplatin) and therefore, a repeat analysis was performed in terms of the patient’s condition. As the patient’s response to chemotherapy was poor, the final consideration was that the condition had not been diagnostically determined subsequent to the multidisciplinary consultation.

**Figure 1:**
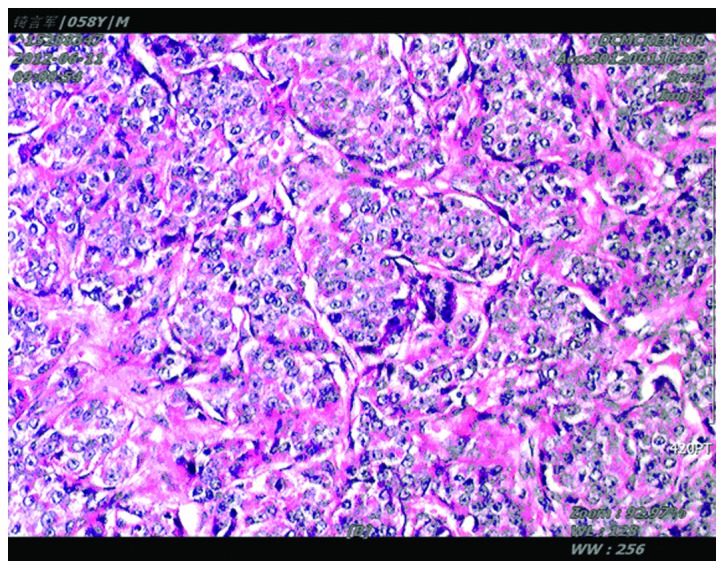
Pathological section for the biopsy of the mass in the left armpit.

Pathological examination is the most reliable diagnostic method (5), therefore, it was recommended that the patient return to the Fujian Union Hospital in order to have the initial pathological section and paraffin blocks sent to Fuzhou General Hospital of Nanjing Military Command for the pathology consultation. The results indicated a poorly-differentiated cancer. Immunohistochemistry analysis of the biopsy revealed the following: Staining for epithelial membrane antigen, E-cadherin, P120, cytokeratin (CK) pan and the estrogen and progesterone receptors was strong (+++), with 90 and 85% positive staining for ER and PR, respectively, while CK7 was weak (+). A Ki-67 of 5% was detected. Thus, immunohistochemistry results of the biopsy specimens of the mass in the left armpit revealed a class I breast invasive ductal carcinoma (Fig. 1).

**Figure 2:**
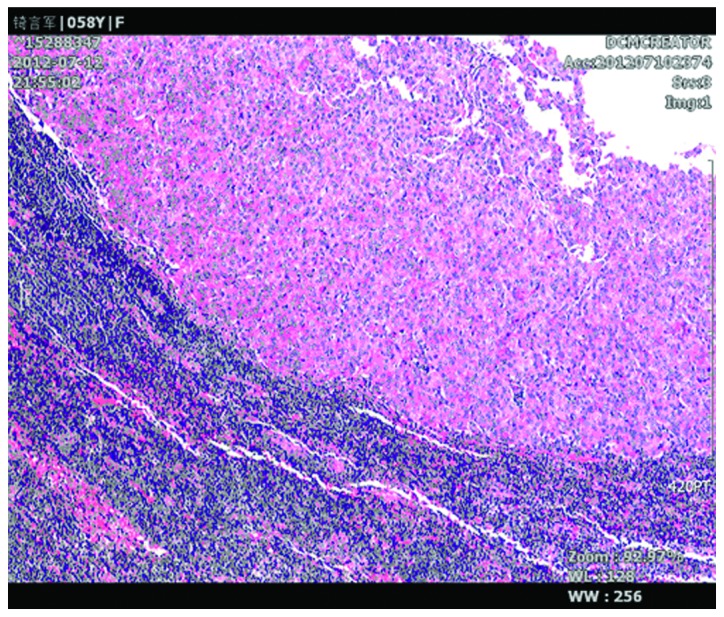
Pathological section of the left mastectomy resection specimen.

The initial diagnosis at the Fujian Union Hospital was correct. Following the determination of the diagnosis as breast cancer with lymph node metastasis, a treatment scheme was proposed. The patient received a left breast cancer modified radical mastectomy in Fuzhou General Hospital of Nanjing Military Command on July 10, 2012. During the surgery, one 10×5×3-cm specimen was resected. According to the pathological examination following the surgery, neither cancer tissue residues nor cancer involving the nipple, skin, breast, basal or skin resection margin were found. No cancer tissue residues were found in the post-operative radical cure specimen of the breast invasive ductal carcinoma. (Fig. 2). The cancer metastasis to the lymph nodes in the armpit was detected. Subsequent to repeated communication concerning the disease, the patient returned to the hospital in September 2012 and received two cycles of doxorubicin hydrochloride (80 mg every three weeks) single-agent post-operative adjuvant chemotherapy and one course of radiotherapy (60 Gy in 30 fractions of 2 Gy per fraction of five fractions per week). According to the follow-ups performed between December 2012 and March 2013, the patient has been able to conduct normal activities, with a markedly improved quality of life, and no further abnormalities have been found.

## Discussion

The cause of MBC is unclear, however, the main risk factors include an increase in the level of estrogen, Klinefelter syndrome along with chromosomal abnormality and gynecomastia (6). The typical clinical manifestation of MBC is a surrounding painless mass, the occurrence rate of which is 75–95% (7). MBC has its own characteristics with regard to onset, risk factors and clinical manifestations, often leading to a delay in the diagnosis and treatment.

No breast mass was found in the physical examination of the patient in the current case. The tumor markers, cancer antigen-125 and carcinoembryonic antigen, were normal. No primary tumors were observed in the mammography and PET-CT, and the patient was diagnosed with breast invasive ductal carcinoma based on the metastases pathology and the immunohistochemical examination, while the primary tumor could not be found following the left breast modified radical mastectomy. However, primary tumors may disappear subsequent to chemotherapy.

The symptoms of MBC are similar to those experiences by females with breast cancer following the menopause. However, the lack of awareness of MBC may delay the diagnosis and treatment, which is likely to result in the progression of the illness. In addition, the majority of cases present with axillary lymph node metastases at diagnosis and are at a late clinical stage. A previous study showed that, according to statistics in 1955, the symptoms prior to diagnosis can be maintained for an average of 21 months (8), while other more recent studies have confirmed that the average delay period for the diagnosis of MBC is between six and 10 months (9). In addition, >40% of MBC are already at phase III/IV at diagnosis (10). Since male breast tissue does not grow, differentiation into a lobule structure is rare, unless the endogenous or exogenous estrogen concentration increases. Therefore, the vast majority of histological MBC types are invasive ductal carcinoma, accounting for >90% of all MBC (2). Due to the late diagnosis and spread of the tumor, the prognosis of MBC is generally worse than that of females with breast cancer. Therefore, the early detection, diagnosis and treatment are key factors for improving the prognosis of MBC. The present study demonstrated that in addition to methods such as clinical features, imaging observations and tumor marker examination, the collection of data through fine-needle aspiration and lumpectomy biopsy in clinical practice are required for evaluation.

Through the diagnosis and treatment of the patient in the present case, the following were confirmed: i) MBC has a low morbidity, often shows clinical manifestations or pathological characteristics that are different compared with common breast cancer, and the primary tumor may be unknown. Therefore a proper analysis should be conducted, with more attention given to such conditions. ii) The pathological report and immunohistochemistry results are extremely important for guiding the diagnosis of malignant tumors. Therefore, imaging diagnostics, such as PET-CT, should not be solely depended on. If the treatment is not effective then the initial diagnosis should be questioned, unless the diagnostic results are absolutely clear. Clinicians should be aware that pathology reports and clinical manifestations should be consistent. iii) Currently, there remains a lack of prospective randomized controlled clinical trial research with regard to MBC treatment. The MBC treatment scheme may also be developed by referencing the experience of female breast cancer treatment, and clinicians should use sufficient medical evidence to prove the scientific rationality of the MBC diagnosis and treatment scheme.

The current study described an extremely rare case of MBC with an unknown primary tumor and highlights a method of the diagnosis and treatment of MBC.
